# Comparison between a laparoscopic articulating needle driver with standard laparoscopic instrumentation for total laparoscopic gastropexy in dogs

**DOI:** 10.1111/vsu.70094

**Published:** 2026-03-05

**Authors:** Erin A. Gibson, William T. N. Culp, Ingrid M. Balsa, Michele Steffey, Philipp Mayhew

**Affiliations:** ^1^ Department of Clinical Sciences and Advanced Medicine University of Pennsylvania Matthew J. Ryan Veterinary Hospital Philadelphia Pennsylvania USA; ^2^ Department of Surgical and Radiological Sciences, School of Veterinary Medicine University of California‐Davis Davis California USA

## Abstract

**Objective:**

To compare the utility of a mechanical laparoscopic articulating needle driver (ALI) to standard laparoscopic instrumentation (SLI) during the creation of a total laparoscopic gastropexy (TLG) in dogs.

**Study design:**

Prospective clinical trial.

**Animals:**

A total of 17 client owned dogs.

**Methods:**

Dogs underwent elective prophylactic TLG and were randomized to ALI or SLI for the first gastropexy suture line. The second line was performed with the alternate instrumentation. Duration of intracorporeal suturing, NASA‐Task Load Index (TLX) questionnaire, modified Instrument Function and Ergonomics survey, and intraoperative incidents were documented per suture line.

**Results:**

Seven female, seven male, two male‐sterilized, and one female‐sterilized dogs were included. Median weight was 38 kg (range: 17.9–66 kg). Four Standard Poodles and German Shepherd dogs each, two Bernese Mountain dogs, and one each of the Airedale, Armenian Gampr, Great Dane, Doberman Pinscher, Anatolian Shepherd, Irish Wolfhound, and mixed breed were enrolled. Median time to complete the suture line with SLI (6 min, range: 5–10) was significantly shorter than ALI (8 min, range: 6–11, *p* < .001). Averaged NASA‐TLX scores of mental demand (*p* = .030), performance (*p* = .014), effort (*p* = .030), and frustration (*p* = .042) were significantly greater for ALI suture lines compared to SLI. Two grade 2 adverse events occurred during TLG.

**Conclusion:**

Suture time and surgeon workload was negatively affected by ALI use compared to SLI. Adverse events were rare and all dogs had TLG successfully completed.

**Clinical significance:**

Both devices can be used to perform TLG. While initial comparison favored SLI, the utility of ALI requires further investigation.

## 1 INTRODUCTION

Gastric dilatation and volvulus (GDV) is a life‐threatening syndrome that occurs when the stomach rotates, causing entrapment of gas, fluid and/or food.^1^, ^2^ This syndrome is most commonly seen in large and giant breed deep‐chested dogs such as Great Danes (39% life‐time risk), Irish Wolfhounds (25% lifetime risk), and Standard Poodles (25% lifetime risk), although GDV can affect any breed of dog.^2^ Gastropexies in large breed dogs are well documented to reduce the risk of GDV later in life.^3^, ^4^, ^5^, ^6^ Prophylactic gastropexy is performed laparoscopically with increasing frequency.^7^, ^8^, ^9^ Total laparoscopic gastropexy (TLG) includes the use of unidirectional barbed suture, a biomechanically sound material,^10^, ^11^ and requires mastery of intracorporeal suturing.^12^, ^13^ Standard laparoscopic needle drivers are rigid instruments that are susceptible to limitations introduced by the fulcrum effect inherent to laparoscopy and limited degrees of freedom.

Flexible needle driver instrumentation has been developed to circumvent the limitations of rigid instrumentation. Such instrumentation offers increased degrees of freedom compared to standard rigid systems, and have been shown to perform comparably or better to rigid instrumentation with improved surgeon ergonomics.^14^, ^15^ Although the addition of multiple degrees of freedom with robotic or mechanical articulating instrumentation is beneficial, the learning curve to utilizing these instruments may be prolonged when compared with standard rigid instruments,^16^, ^17^, ^18^, ^19^ which is a possible downside and should be considered when incorporating these instruments in practice or during comparison to standard laparoscopic instrumentation.

The FlexDex needle driver (FlexDex Surgical, Inc., Brighton, Michigan), is a mechanical articulating needle driver offering enhanced dexterity for intracorporeal suturing.^19^ This was introduced in 2017 and compared to standard instrumentation, the articulating needle driver (ALI) has demonstrated improved ergonomics and effectiveness when suturing in challenging anatomic locations.^19^ Recommendations prior to use include adequate training and the availability of three‐dimensional (3D) imaging systems.^18^, ^20^, ^21^, ^22^ Clinical experience with this instrument is limited and a single report exists in veterinary medicine describing a steep learning curve for it's use for laparoscopic gastropexy, and success in completing TLG.^23^ In human patients, clinical outcomes and surgeon ergonomics appear to be improved relative to rigid instruments.^20^, ^24^ Currently, no study exists comparing utility of a mechanical hand‐held articulating needle drive and standard laparoscopic instrumentation in laparoscopic gastropexy.

The objectives of this early feasibility trial was to perform TLG with a mechanical hand‐held ALI and standard laparoscopic instrumentation (SLI) and compare surgical time, NASA‐TLX score, modified Instrument Function and Ergonomics survey, and modified Objective Structured Assessment of Technical Skills/Global Components Score (OSATS/GCS) between the two groups. The authors hypothesized that the use of a mechanical ALI would result in comparable surgical time, decreased mental workload and improved surgeon ergonomics compared to SLI.

## 2 MATERIALS AND METHODS

### 2.1 Animals

Client‐owned dogs presenting for elective TLG between 04/2022–05/2023 were eligible for prospective enrollment in the study. Dogs undergoing concurrent gonadectomy were included as long as the planned TLG was performed first. Eligibility for elective surgery was determined by the primary investigator based on patient history, physical examination, and clinical laboratory findings. Dogs less than 6 years of age at enrollment had minimum database performed (blood glucose, packed cell volume, total solids, blood urea nitrogen) while dogs older than 6 years of age at the time of enrollment, had complete blood counts as well and a comprehensive, standard biochemical profile performed. The protocol was approved by the Clinical Trials Review Board (IACUC protocol # 22680). The study protocol was discussed with owners of all dogs and written informed consent was obtained prior to enrollment.

### 2.2 Total laparoscopic gastropexy procedure

All surgeries were performed by a single board‐certified surgeon experienced with laparoscopy, and all surgeries were performed using the 10 mm TIPCAM1 S 3D, 0° (Karl Storz Veterinary Endoscopy, Goleta, California) in 3D mode. All dogs were placed under general anesthesia and monitored by the Small Animal Clinical Anesthesia Service; anesthetic protocols were determined/approved by the attending anesthetist. Dogs were placed in dorsal recumbency with the tower and monitor on the dog's right side, and surgeon on the dog's left side. The TLG was performed as previously reported, with some modifications.^12^ Briefly, a modified‐Hasson technique was performed to place the first 6 mm port in the subumbilical position. The second 12 mm port was placed approximately 5 cm cranial to the subumbilical port and the cranial‐most third port was placed approximately 5 cm cranial to the middle port. The middle port was used to accommodate the 3D telescope, and laparoscopic instrumentation placed through the cranial‐most and caudal‐most ports. After visualizing the stomach, a percutaneously placed transabdominal suspension suture of 0 monofilament on a 36 mm ½ c needle was passed through the body wall in the right cranial quadrant, and subsequently through the pyloric antrum, approximately 4 cm orad to the pylorus in the region of the planned TLG, in order to suspend the stomach from the body wall, 2–5 cm caudal to the last rib and 5–7 cm lateral to the right of midline. Following this, the transversus abdominis muscle was incised with a j‐hook monopolar electrosurgical instrument in a direction that was perpendicular to slightly oblique to the orientation of the muscle fibers. Subsequently, the seromuscular layer of the stomach was scored using the monopolar probe over a 4 cm length of the pyloric antrum. A 15‐cm length of 2–0 monofilament absorbable barbed suture (V‐Loc 180; Medtronic Inc., Salem, Massachusetts) was passed into the peritoneal cavity percutaneously and used to appose the lateral most aspect of the transversus incision to the incised seromuscularis edge closest to the greater curvature in a simple continuous pattern. The first suture line was performed with either SLI or ALI based on randomization in a 1:1 ratio using a simple randomization sequence generated using an online randomization tool (randomizer.org). Suturing was performed with the surgeon's right hand, in a right to left direction. The suture line was completed with two additional perpendicular passes relative to the suture line in the transversus muscle, and a final back‐handed bite parallel to the suture line to cross the final two passes and secure the barbed suture. The barbed suture was cut intracorporeally while leaving a 1 cm tag and the needle with remaining suture passed through the body wall for removal.

Following this, the surgeon filled out the NASA‐TLX^25^ and modified Instrument and Ergonomics surveys (Appendix S1).^18^ Following completion of these surveys, the suturing procedure was repeated to appose the medial incised edge of the transversus abdominis to the incised edge of the seromuscularis layer of the stomach nearest to the lesser curvature with the alternative needle driver device compared to what was used for the first line, in a right to left direction as well. Once the second suture line was completed as described above, the suture was cut and needle passed through the abdominal wall as described above. The suspension suture was removed, and the NASA‐TLX and modified Instrument and Ergonomics survey were completed for the 2nd instrument/line.

### 2.3 Data collection and questionnaires

Duration of the TLG procedure was measured by the operating room technician from time of first grasp of the unidirectional barbed suture needle intracorporeally, to the time following the passage of the needle through the body wall following final pass through the transversus abdominus following completion of the second suture line. Duration of intracorporeal suturing per side (far side vs. near side) was defined as the time from first grasp of the unidirectional barbed suture needle intracorporeally, to the time following passage of the needle through the body wall following final pass through the transversus abdominus. Total gastropexy time was time from first incision until closure of port‐sites, while excluding the time taken to complete surveys. All intraoperative complications were determined by the surgeon and recorded by the operating room technician. Immediately following completion of each suture line, the primary surgeon completed the NASA‐TLX questionnaire and the Instrument Function and Ergonomics survey (Appendix S1).^18^, ^26^ Videos taken of both ALI and SLI suture lines were randomized and assigned to two laparoscopic suturing experts (ACVS Fellows of Minimally Invasive Surgery) for grading according to the modified Objective Structured Assessment of Technical Skills/Global Components Score (OSATS/GCS) laparoscopic suturing evaluation system (Appendix S1) that has been previously validated in assessing quality of laparoscopic suturing.^27^, ^28^, ^29^ Global rating checklist (GRC) scores (Appendix S2) were summed per suture line by each grader.

The primary operating surgeon did not have experience with ALI before clinical trial initiation but was proficient in TLG with SLI. Prior to trial enrollment, simulator training was performed by the primary surgeon under direct vision. These included training tasks provided by the manufacturer such as intracorporeal suturing with suture pads and ring transfer. The authors defined proficiency for ring transfer as successful completion of the task under 60 s with no drops outside the field of view and completion of intracorporeal suturing task in under 112 s. Proficiency by the primary surgeon in all tasks was achieved prior to proceeding with clinical cases in order to avoid obvious biases that could be present when using newer devices which the operating surgeon has less experience with and training began 3 months prior to case enrollment. Task repetition was not dictated, and hours spent in simulation training was approximately 1 h/week and was left to the discretion of the primary surgeon. Task performance was not consistently evaluated prior to each procedure once trial enrollment began.

### 2.4 Adverse events (AEs)

The previously published CLASSIC intraoperative adverse events surgery was used to classify intraoperative errors.^30^ The Clavien Dindo Classification of Surgical Complications was applied to all postoperative adverse events.^31^

### 2.5 Statistical analysis

Multiple endpoints were assessed in an exploratory manner without formal adjustment for multiplicity, so *p*‐values are interpreted cautiously. Outcomes measure scores (NASA‐TLX, modified OSATS/GCS, modified Instrument Function and Ergonomics survey) between techniques were compared using a paired *t*‐test or non‐parametric equivalent or with linear mixed models.

Normality of continuous variables was assessed with the Shapiro–Wilk test and continuous variables reported as median and range or mean and SD for non‐normal and normally distributed data, respectively. Differences of mean or median values between treatment groups were determined by paired *t*‐tests or Wilcoxon matched pairs signed rank tests, respectively. Significance was defined as *p* < .05.

Dogs were assigned by use of a randomization scheme to either ALI or SLI for the first suture line. Randomization was in a 1:1 ratio using a simple randomization sequence generated using an online randomization tool (randomizer.org). Treatment allocations were concealed in sequentially numbered opaque sealed envelopes and opened after enrollment by a single investigator responsible for the TLG.

The effect of ALI on total and component NASA‐TLX, ergonomic, and GRC scores and suture times were analyzed with mixed effect models and an independent covariance structure with dog as the random intercept and treatment (i.e., the ALI vs. SLI device), sequence (i.e., order of devices as either ALI first then SLI second or SLI first then ALI second) and period (i.e., the first vs. second instance of device use) as fixed effects. Agreement between OSATS/GCS graders was determined by calculating the intraclass coefficient (*r*) using a two‐way random effects model with values of *r* < .5, between .5 and .75, between .75 and .9 and >.9 as indicating poor, moderate, good, and excellent agreement, respectively.^32^

## 3 RESULTS

### 3.1 Animals

A total of 17 client owned dogs (7 females, 7 males, 2 male‐sterilized, and 1 female‐sterilized) that were presented for prophylactic TLG were enrolled in the study. Median weight was 38 kg (range: 17.9–66 kg) and median age was 10 months (range: 10–76 months). Dog breeds included 4 Standard Poodles and German Shepherds each, two Bernese Mountain dogs, and one each of the Airedale Terrier, Armenian Gampr, Great Dane, Doberman Pinscher, Anatolian Shepherd dog, Irish Wolfhound, and Great Pyrenees‐German Shepherd mix.

### 3.2 Procedures

Nine dogs were randomized to receive ALI instrumentation first while eight dogs received SLI first. A total of 11 dogs had concurrent procedures performed following TLG including ovariectomy (*n* = 7 dogs), prescrotal neuter (2), intra‐abdominal and inguinal cryptorchidectomy (1), and umbilical hernia repair (1). Median TLG time was 30 min (range: 15–55 min). Median time to complete the suture line with SLI was 6 min (range: 5–10 min) and with ALI was 8 min (range: 6–11 min). Adverse events during TLG included two grade 2 events (failure of the SLI device requiring replacement). No postoperative complications occurred.

### 3.3 NASA‐TLX scores

Individual NASA‐TLX component scores of mental demand, performance, effort, and frustration were significantly greater for ALI suture lines compared to SLI regardless of whether the scored suture line was performed first or second (period), or the order ALI suturing was performed relative to SLI (sequence) (Table 1). Performance and frustration scores were significantly affected by period, meaning scores were significantly higher for the second suture line for frustration (*p* = .021) and performance (*p* = .025) correlating with greater frustration and poorer perceived performance, regardless of what device (ALI or SLI) was used. Per individual score, regardless of device used, period effect of the second suture line added a mean score of 26 (95% CI: 4–50, *p* = .021) for frustration, and a mean of 25 (95% CI: 5–44, *p* = .025) for performance.

**TABLE 1:** NASA‐task load index subcategory scores using SLI and the effect of using a mechanical ALI associated with suturing.

	Baseline score using SLI^a^	Effect of ALI on baseline Score^c^ (95% CI)	*p* value^b^
Mental demand (95% CI)	118 (66–170)	+40 (4–75)	.030^b^
Physical demand (95% CI)	34 (22–45)	+2 (−6 to −11)	.61
Temporal demand (95% CI)	32 (21–43)	+0.7 (−4.5 to 5.9)	.78
Performance (95% CI)	110 (82–138)	+25 (5–44)	.014^b^
Effort (95% CI)	66 (42–90)	+17 (2–32)	.030^b^
Frustration (95% CI)	75 (40–110)	+24 (1–46)	.042^b^

Abbreviations: ALI, articulating needle driver; SLI, standard laparoscopic instrumentation.

^a^ Scores for SLI suture lines in the SLI then ALI sequence.

^b^ *p*‐value <.05 is significant.

^c^ Measured change in score due ALI compared to SLI.

### 3.4 Suture time

Only treatment (ALI vs. SLI) was identified to have a significant impact on suture time, with suture lines performed with ALI significantly slower compared to suture lines performed with SLI (Table 2). Overall, the effect of treatment (use of the ALI device) contributed a mean of 1.9 min (95% CI: 1.2–2.6, *p* < .0001) to the time it took to complete a suture line of the TLG.

**TABLE 2:** Suture time in minutes per mechanical ALI or SLI device in reference to suture line sequence and period.

Treatment sequence^b^	Treatment period^a^	
	1	2
ALI than SLI	ALI	SLI
Mean (95% CI)	8.0 (7.0–9.0)	6.2 (5.2–7.2)
Sample size	9	9
SLI then ALI	SLI	ALI
Mean (95% CI)	6.3 (5.2–7.3)	8.4 (7.3–9.4)
Sample size	8	8

Abbreviations: ALI, articulating needle driver; SLI, standard laparoscopic instrumentation.

^a^ Treatment period represents the use SLI or ALI as first suture line device or second suture line device.

^b^ Treatment sequence represents the combined order of use of SLI and ALI devices for completion of total laparoscopic gastropexy.

### 3.5 Instrument function and ergonomics survey

No significant association was identified between instrument function and ergonomic scores and any of the assessed factors (Table 3). Interestingly, forearm score tended to be lower in the second suture line, regardless of device used, by an average of −0.28, (*p* = .088) although this was not found to be significant.

**TABLE 3:** Ergonomic scores recorded by the surgeon immediately following suture lines completed with the ALI or SLI device.

Ergonomic component	Constant (SLI)^a^	Treatment effect^b^	*P* value^c^
Shoulder (95% CI)	2.3 (1.8–2.7)	0.08 (−0.4–0.6)	.76
Neck (95% CI)	3.6 (3.2–4.1)	−0.11 (−0.35–0.12)	.35
Back (95% CI)	4.0 (3.9–4.1)	−0.06 (−0.18–0.05)	.29
Wrist (95% CI)	2.8 (2.4–3.1)	−0.25 (−0.6–0.1)	.16
Forearm (95% CI)	2.8 (2.4–3.1)	0.16 (−0.17–0.49)	.34
Hand (95% CI)	3.1 (2.6–3.6)	−0.007 (−0.39–0.38)	.97

Abbreviations: ALI, articulating needle driver; SLI, standard laparoscopic instrumentation.

^a^ Scores for SLI suture lines in the SLI then ALI sequence.

^b^ Measured change in score due ALI compared to SLI.

^c^ *p*‐value <.05 is significant.

### 3.6 Global rating checklist

**FIGURE 1:**
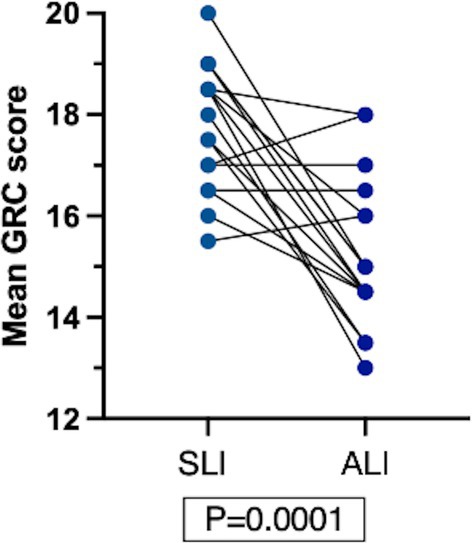
Mean global rating checklist (GRC) scores for suture lines performed with standard laparoscopic instrumentation (SLI) compared to suture lines performed with mechanical laparoscopic articulating needle driver (ALI) in dogs undergoing laparoscopic gastropexy.

GRC scores were completed for all videos by both investigators. Mean GRC scores for all dogs were calculated for SLI (17.7, SD 1.24) and ALI (15.24 SD 1.48) with a maximum possible score of 20 (Appendix S2). Paired *t*‐tests to compare the scores identified significantly higher scores for SLI compared to ALI (*p* < .0001, Figure 1). Additionally, when the ALI device was used as the second device rather than the first, there was a more substantial decrease in GRC scores, correlating with poorer observed performance (*p* = .021). Sequence of device use (ALI then SLI or SLI then ALI) alone did not lead to significant differences in scores, and significant difference in scores was only observed when evaluating ALI device sequence. Inter‐rater agreement per suture line for GRC was found to be moderate to low (*r* = .36), defined as a grade less than *r* = .5.

## 4 DISCUSSION

ALI was found to be a safe and effective alternative to SLI for TLG in dogs, although in this early feasibility study did not provide obvious advantages compared to the SLI device for the completion of laparoscopic gastropexy. ALI instrumentation also appeared to significantly increase surgeon mental workload despite working in a 3D environment. Despite these findings, all dogs had TLG completed without requiring conversion. Adverse events were infrequent and low grade which was an acceptable outcome.

In this study, mental workload estimated by certain individual NASA‐TLX components (frustration, performance, effort, mental demand) appeared to be greater for ALI device usage relative to SLI device usage. Surgical time was minimally (1–2 min) longer in the ALI group, which is of limited clinical significance, despite being significant statistically. In healthy dogs undergoing elective TLG, it is unlikely that a 1–2 min difference in surgical time would alter patient outcomes which was supported by the excellent outcomes of all dogs in this cohort. This gap could be accounted for by less familiarity with the ALI device relative to the SLI. Prior studies have identified advantages of multiple degrees of freedom with hand‐held articulating instrumentation, although this can be offset by an initial learning curve.^22^, ^33^ This appears to be true in clinical application of articulating instruments as well.^24^ The learning curve for use of articulating instrumentation compared to rigid instrumentation in task performance is dependent on various factors such as user experience and is difficult to generalize.^33^, ^34^ While some studies have documented non‐inferior operative times,^35^, ^36^ longer operative time for procedure or task completion using articulating instrumentation has also been documented in human medicine.^17^, ^18^, ^37^ A recent study by Massari et al.^23^ evaluating the learning curve for a handheld robotic laparoscopic needle driver in completing laparoscopic gastropexy in dogs documented a steep learning curve. In this study, two‐port laparoscopic gastropexy with a handheld robotic laparoscopic needle driver was performed in all cases and learning curve assessments were based on surgical time. Plateauing surgical time occurred at the 12th case. These findings serve to potentially enhance the findings of our study, which is that ALI‐specific results may reflect an initial lack of expertise, considering our cohort of 17 dogs would have minimally surpassed the learning curve suggested by Massari et al.^23^

To minimize any effect the learning curve had on ALI performance, a series of tasks with endpoint criteria, to be completed on ALI device by the operating investigator was assigned. Despite this, it remains possible that the diminished performance of the ALI device is explained by the slope of the learning curve prior to plateauing into expertise. Additionally, only 17 clinical uses of the ALI device were performed over the span of approximately 1 year, which could explain a longer learning curve and failure to reach “expertise” during the course of this study. Cautious interpretation of the results is important prior to dismissing the ALI device.

3D imaging was used in this study to optimally emulate a robotic surgical environment. Prior research in veterinary laparoscopy has identified surgeon workload benefits in using 3D imaging systems for laparoscopic gastropexy.^12^ Additionally, two‐ and 3D imaging has been assessed in task performance with the articulating needle driver used in this study, with 3D imaging enhancing task performance.^22^ The utility of 3D imaging was not directly assessed in this study although ultimately provided internal consistency. It is important to keep in mind however that results of this study may have limited applicability in surgical centers that do not have access or routinely use a 3D imaging system.

Occupational injury secondary to laparoscopic surgery occurs with relative frequency in human medicine.^38^ Maximizing comfort and mitigating injury remain a priority and are important considerations in veterinary laparoscopy. Handheld articulating devices have demonstrated improved ergonomics based on diminished range of motion of the elbow, shoulder, and wrist joints compared to standard rigid devices in some studies.^14^, ^24^ In this study, ergonomic scores, representing physical workload, were not significantly different between SLI and ALI. The duration of use for both instruments was relatively short (typically less than 10 min), which may have prevented the surgeon from reliably distinguishing a physical advantage. Finally, the cohort of enrolled dogs varied widely in size. It is possible that our port placement was not optimized for the heterogenous body conformations that were encountered during this study, which could have affected ergonomic scores. More cases of a uniform size and ongoing use of devices such as the ALI may be necessary to better understand physical ramifications.

While the ALI device appeared to perform less well compared to SLI in some aspects, the investigators found the added dexterity and multiple degrees of freedom was subjectively advantageous. Clinically, the ability to manipulate the needle driver to remain perpendicular to the incision during intracorporeal suturing was helpful. While this was not specifically measured in this study, prior studies identified this as a reliable advantage of needle drivers with multiple degrees of freedom compared to SLI.^39^, ^40^ This was slightly offset by the decreased perceived needle grasping strength compared to SLI by the operating surgeon in this study. Clinically, this translated to challenges with passing the needle through wider swaths of tissue such as transversus abdominis of the abdomen and seromuscular layers of the stomach with a large needle and barbed suture, which had increased tissue drag compared to standard monofilament. Using the ALI device in other minimally invasive procedures may identify a more obvious benefit of ALI compared to SLI beyond TLG.

Video GRC scores identified significantly lower scores, correlating with poorer performance, for the ALI device compared to SLI, regardless of sequence or period. Additionally, scores were further affected by period for the ALI device, meaning that use of the ALI device for the second suture line correlated with poorer performance compared to using the ALI device for the first suture line. This somewhat correlates to the independent NASA‐TLX scores of performance and frustration, which were significantly higher for the second suture line, correlating with greater frustration and poorer perceived performance, regardless of what device (ALI or SLI) was used. It is possible that cumulative physical or mental fatigue may have contributed to diminished success in the second suture line and may have been magnified during use of a less familiar instrument (ALI). While not standardized to this study, the authors suggest that performing warm‐up tasks with the ALI device could have improved procedural performance and may have mitigated some of the diminished performance over time. Preoperative warm‐up exercises before laparoscopic procedures have been correlated to improved surgeon performance in some studies.^41^, ^42^ The relative infrequency of these procedures during the data collection period may have prolonged the learning curve with this device, and warm‐up exercises could be considered prior to using this device in the future.

Limitations of this study include the small number of dogs enrolled which prevents definitive conclusions from being drawn regarding utility of this device. Additionally, task workload was self‐reported and additional objective measures of physical workload such as EMG^43^ was not performed. These results reflect the experience of a single surgeon, and likely additional pooled data collected over a greater number of cases and multiple surgeons could enhance understanding of ALI device utility. It is also possible that more obvious utility of the ALI device would be observed in more traditionally challenging spaces or orientations to suture, such as the vertical or y‐axis, encountered in procedures such as diaphragmatic hernia repair. In short, laparoscopic gastropexy in a heterogenous group of large breed healthy dogs may limit obvious utility that could be observed with the ALI. Additionally, the ALI device was reused until an overt breakdown of the device occurred and was based on the operating surgeon's preference. It is possible that subtle diminished function occurred prior to overt breakage, which may have altered ALI device performance.

In conclusion, the ALI device was associated with greater surgical time, increased surgeon workload, and decreased proficiency compared to the SLI device. However, the differences in performance appeared to be clinically insignificant based on the successful completion of TLG in all dogs, with all devices, and minimal adverse events reported. These findings represent early feasibility based on a single center single operator experience with this device, and diminished performance could be related to unfamiliarity with the device compared to SLI. The investigators noticed improved dexterity with the needle driver although concerns with needle grasper strength were also observed. Acknowledging the limitations of this study, further investigation into the utility of the ALI device in veterinary laparoscopy and thoracoscopy is warranted. ALI is a safe and effective device to complete TLG in large‐ to giant‐breed dogs.

## AUTHOR CONTRIBUTIONS

Gibson EA, DVM, DACVS (Small Animal): Co‐conceived study, performed surgical procedures, data collection, and manuscript preparation. Culp WTN, VMD, DACVS (Small Animal): Co‐conceived study, data collection and manuscript preparation. Balsa IM, MEd, DMV, DACVS (Small Animal): Data collection and manuscript preparation. Mayhew P, BVM&S, MRCVS, DACVS (Small Animal): Data collection and manuscript preparation. Steffey M, DVM, DACVS (Small Animal): Data collection and manuscript preparation.

## FUNDING INFORMATION

Grant funding (2021‐51‐F) provided by the Center for Companion Animal Health, University of California (UC) Davis School of Veterinary Medicine.

## CONFLICT OF INTEREST STATEMENT

The authors declare no conflict of interested related to this report.

## Supporting information


**Appendix S1.** Supporting Information.

## Data Availability

The data that support the findings of this study are available from the corresponding author upon reasonable request.
