# Supplementary material for: Comparison between a laparoscopic articulating needle driver with standard laparoscopic instrumentation for total laparoscopic gastropexy in dogs
Source: Vet Surg. 2026 Mar 5;55(6):1113–21. doi: 10.1111/vsu.70094 (PMC13420948; doi:10.1111/vsu.70094)
Supplement: Supplementary file 1 — Appendix S1. Supporting Information. [file VSU-55-1113-s001.docx]

**APPENDICES**

**Appendix 1**


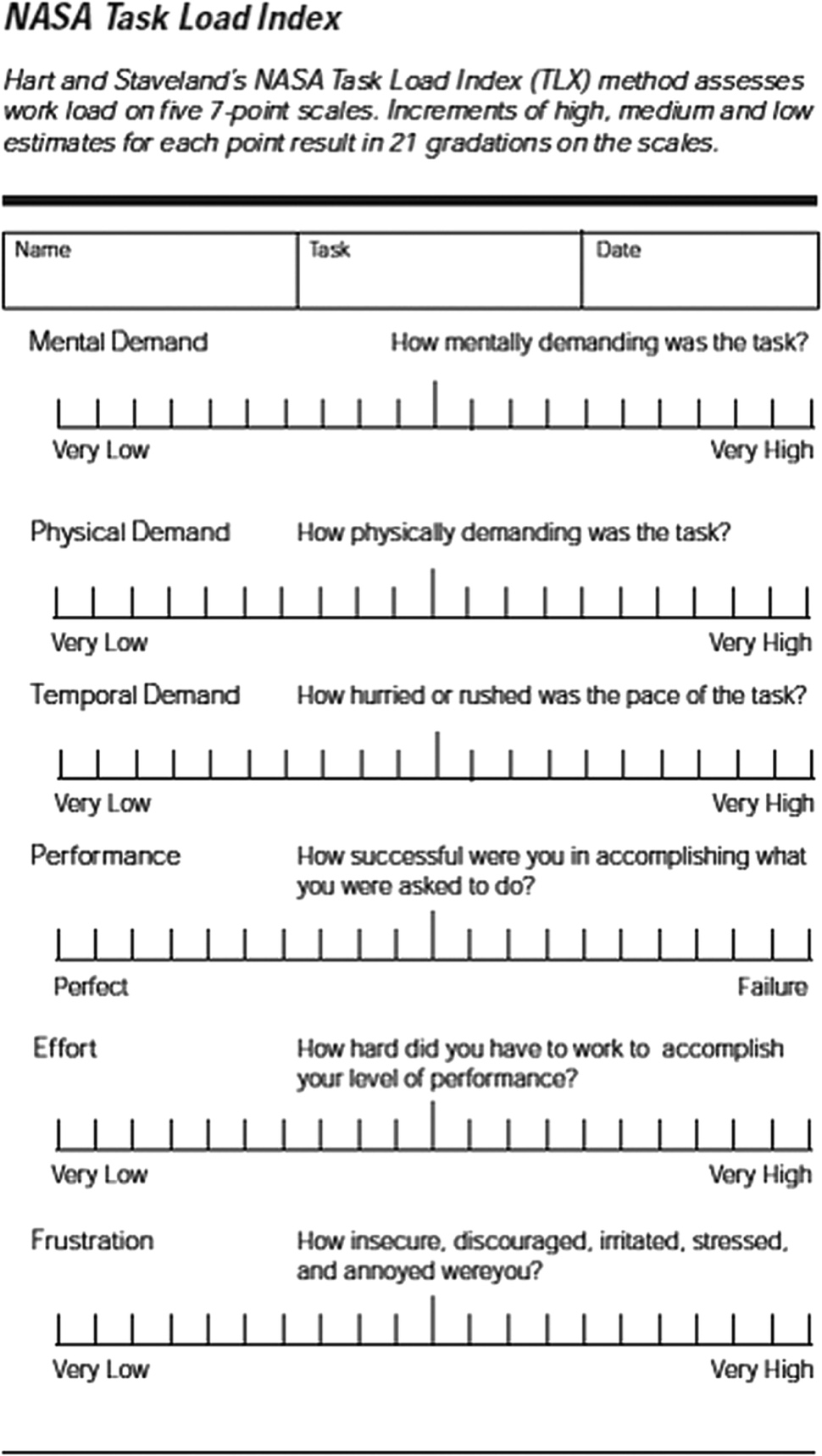


**From: https://humansystems.arc.nasa.gov/groups/tlx/downloads/TLXScale.pdf**

**Instrument Comfort/Ergonomics Survey**

**Please place a mark in the following box that corresponds to your level of strain or discomfort while using the instrument (corresponding side of instrument use).**

|  | High Strain (1) | Moderate Strain (2) | Mild Strain (3) | No Strain (4) |
| --- | --- | --- | --- | --- |
| Shoulder |  |  |  |  |
| Neck |  |  |  |  |
| Back |  |  |  |  |
| Wrist |  |  |  |  |
| Forearm |  |  |  |  |
| Hand |  |  |  |  |
|  |  |  |  |  |

Modified from: Criss CN, Jarboe MD, Claflin J, Matusko N, Rooney DM. Evaluating a Solely Mechanical Articulating Laparoscopic Device: A Prospective Randomized Crossover Study. *J Laparoendosc Adv Surg Tech A*. 2019;29(4):542-550. doi:10.1089/lap.2018.0539

**Appendix 2**

**Global Rating Checklist (GRC)**

| **Tissue and Instrument Handling** | | | | |
| --- | --- | --- | --- | --- |
| 1  Rough movement; awkward handling of instruments and tissue (or model) | 2 | 3  Careful handling of instruments and tissue (or model) overall, with occasional awkward movements | 4 | 5  Consistently appropriate and careful handling of instrumens and tissue (or model) |
| **Depth Perception/Accuracy** | | | | |
| 1  Constantly misses target, slow to correct | 2 | 3  Sometimes misses target, quick to correct | 4 | 5  Accurately direct instrument to target |
| **Dexterity/Efficiency** | | | | |
| 1  Uncertain, inefficient movements without progress | 2 | 3  Efficient movements overall with some unnecessary moves | 4 | 5  Fluid, efficient movmeents without wasted time or motion |
| **Autonomy (Proficiency)** | | | | |
| 1  Unable to complete entire task at this time | 2 | 3  Able to perform task safely with some instruction | 4 | 5  Able to perform task safely and independently |

**Total Score:_______/ 20 points total.**

Modified from: Vassiliou MC, Feldman LS, Andrew CG, Bergman S, Leffondré K, Stanbridge D, Fried GM. A global assessment tool for evaluation of intraoperative laparoscopic skills. Am J Surg. 2005 Jul;190(1):107-13.
